# The Health and Economic Effects of PCV15 and PCV20 During the First Year of Life in the US

**DOI:** 10.3390/vaccines12111279

**Published:** 2024-11-14

**Authors:** Aleksandar Ilic, Maria J. Tort, Alejandro Cane, Raymond A. Farkouh, Mark H. Rozenbaum

**Affiliations:** 1 Pfizer Inc., Tadworth KT20 7NY, UK; 2 Pfizer Inc., Collegeville, PA 19426, USA; 3 Pfizer Inc., 2909 LD Capelle aan den Ijssel, The Netherlands

**Keywords:** pneumococcal conjugate vaccine (PCV), PCV20, PCV15, invasive pneumococcal disease (IPD), cost-effectiveness, vaccine effectiveness, vaccine

## Abstract

(1) Background/Objectives: Two pneumococcal conjugate vaccines, 15-(PCV15) and 20-(PCV20) valent formulations, are routinely recommended for US children in a 3+1 schedule. The first three doses are administered during the first year of life at 2, 4, and 6 months, while a booster dose is given at 12 to 15 months. This study evaluated the health and economic effects of the PCV20 infant series within the first year of life compared to PCV15. (2) Methods: Using a decision-analytic model, we calculated the health and economic effects of introducing PCV15 or PCV20 for five subsequent birth cohorts. Epidemiological data were drawn from peer-reviewed studies and estimates for vaccine effectiveness were extrapolated from established PCV13 effectiveness and PCV7 efficacy studies. Direct medical costs related to the disease treatment were extracted from the literature and inflated to 2024 dollars. (3) Results: Over the course of five years, the implementation of PCV20 vaccination for newborns in the United States, compared to PCV15, is projected to prevent an additional 220 cases of invasive pneumococcal disease, 6542 cases of community-acquired pneumonia, and 112,095 cases of otitis media within the first year of life across five subsequent birth cohorts. This strategy could prevent 66 infant deaths linked to these illnesses and confer extra health gains, amounting to 5058 years of life and 5037 quality-adjusted life years. These prevented cases are estimated to save approximately USD 147 million over 5 years. (4) Conclusions: This study demonstrated that vaccinating with PCV20 during the first 12 months of life compared to PCV15 in the US would yield a substantially greater health and economic return due to the five additional serotypes covered by PCV20.

## 1. Introduction

*Streptococcus pneumoniae* is a major cause of illness and death among children and adults, with vaccination being the key method for preventing pneumococcal infections [1]. The introduction of the 7-valent pneumococcal conjugate vaccine (PCV7) (comprising the serotypes 4, 6B, 9V, 14, 18C, 19F, and 23F) in 2000 resulted in significant decreases in invasive pneumococcal disease (IPD), community-acquired pneumonia (CAP), and otitis media (OM) while also benefiting unvaccinated populations through indirect protection [1,2,3]. In 2010, the 13-valent pneumococcal conjugate vaccine (PCV13) (which includes serotypes 1, 3, 5, 6A, 7F, and 19A, in addition to PCV7) led to further declines in pneumococcal disease [4,5,6,7,8,9].

Currently, two PCVs, PCV15 and PCV20, are recommended for US infants [10,11]. PCV15 comprises two additional serotypes (22F and 33F) compared to PCV13, while PCV20 comprises five additional serotypes (8, 10A, 12F, 15B, 18B and 19A) compared to PCV15 [10]. These vaccines are given in a 3+1 schedule in the US, with the first three doses being administered at 2, 4, and 6 months, followed by a booster dose given between 12 and 15 months [12].

Previously published cost-effectiveness studies have shown that pediatric use of PCV20 could yield substantive reductions in the clinical burden of pneumococcal disease when compared to PCV13 and PCV15 at the population level [13,14,15,16,17,18,19,20,21,22]. For example, a recent US study showed that PCV20, compared to PCV15, could prevent more than 29,633 cases of IPD, 252,746 cases of all-cause hospitalized non-bacteremic pneumonia (NBP), 1.1 million cases of all-cause ambulatory NBP, 2.7 million cases of all-cause OM, and 9737 deaths associated with these diseases among the US population over a 10-year period [13]. Notably, a substantial portion of these health benefits would accrue in adults and the elderly due to indirect effects.

Currently, no modeling studies have specifically looked at the health and economic effects of PCV15 or PCV20 during the first year of life, when children are at the highest risk of pneumococcal infections. During 2019 (the last pre-COVID19 year) in the US, IPD incidence was approximately 32% higher in the first year of life compared to the second year of life, while IPD and hospitalized pneumonia mortalities among children in the first year of life were approximately 32% and 260% higher, respectively, compared to children in the second year of life [13,23,24].

Protecting infants was the main reason for introducing routine infant pneumococcal vaccination, considering their vulnerability to pneumococcal infections and the potential for severe outcomes. Therefore, our study specifically examines the health and economic effects of a 3-dose infant series of PCV15 and PCV20 within the first year of life. By doing so, we intend to isolate the direct benefits of pre-booster vaccination, providing a clearer understanding of the effects of the infant vaccination series without the confounding influence of herd effects, which typically develops over a longer period as vaccination coverage in the population increases. This approach allows us to focus on the immediate and direct health outcomes for infants who are the primary recipients of these vaccines and underscores the significance of timely vaccination in the first year of life.

## 2. Materials and Methods

### 2.1. Model Description

**Figure 1:**
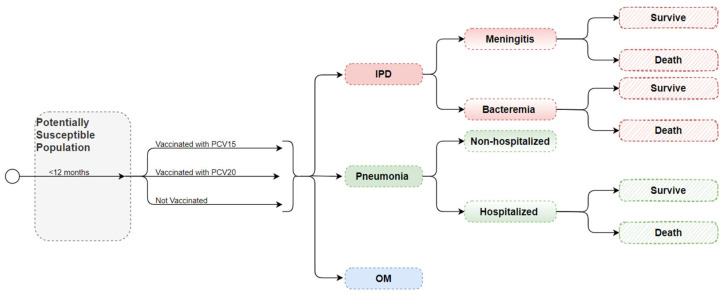
Model schematic.

Using a decision-analytic multi-cohort model, we calculated the health and economic effects of PCV15 and PCV20 for infants within the first year of life for 5 subsequent birth cohorts (Figure 1). Infants born into the model were subject to vaccine uptake assumptions and, if vaccinated, were assumed to receive the 3 doses administered at 2, 4, and 6 months [12,25]. Each annual birth cohort was followed for a single year, after which they were removed from the model.

Vaccine uptake was based on estimates from the National Immunization Survey, which is a telephone survey used to monitor vaccination coverage among children and teens [25]. These data suggest that the coverage of PCV13 when all three doses of the vaccine are completed by 13 months of age was 87.5% in the 2021 cohort, which informed our model for 2022–2027.

Clinical outcomes and economic costs were projected for 5 US birth cohorts, beginning in 2022 (n_1_ = 3.61 M; n_2_ = 3.55 M; n_3_ = 3.49 M; n_4_ = 3.43 M; and n_5_ = 3.37 M) [13]. Clinical outcomes included (1) cases of IPD, stratified by clinical manifestation into meningitis and bacteremia; (2) cases of CAP, stratified by care setting into hospitalized and non-hospitalized patients; (3) cases of all-cause OM; (4) disease related deaths; (5) life years (LYs); and (6) quality-adjusted life years (QALYs). Economic costs included costs averted (per disease and total), which were generated based on projected clinical outcomes and corresponding direct unit costs. All costs were inflated to 2024 US dollars [26].

Our analysis was performed from a healthcare perspective, focusing on direct medical costs and health outcomes related to the vaccines, excluding indirect costs such as caregiver productivity losses or broader societal impacts. The analyses were carried out in Microsoft Excel, version 365.

Recognizing that certain health outcomes manifest over a longer timeframe than the time horizon used in our model, we projected QALYs and LYs gained across the expected lifespan of the infants, similar to other studies [13,14].

### 2.2. Model Estimation

Input parameters were estimated based on the data from peer-reviewed studies and published sources. These parameter values are summarized in Table 1.

**Disease incidence rates.** IPD incidence was based on a 2019 (last year prior to onset of COVID-19, used to avoid disruptions) report from the Active Bacterial Core Surveillance System (ABCs) [23]. Incidence rates in 2019 (13.7 per 100,000 for children less than 1 year) were similar to the observed rates of the prior 3 years (11.6–13.3 per 100,000) [36]. The percentage of IPD cases which manifested as meningitis was assumed to be 16% based on the study by Prasad et al. (2023), while the remaining IPD cases were assumed to manifest as bacteremia [24].

Hospitalized CAP incidence rates were based on the all-cause inpatient pneumonia rates, estimated from the 2018 to 2019 averages in the National Inpatient Sample (NIS), as reported in the study by Prasad et al. (2023) [24]. All-cause non-hospitalized pneumonia incidence rates were based on Tong et al. (2018), who retrospectively assessed data from the MarketScan^®^ Commercial Claims and Encounters database to determine the frequency of pneumonia in the US from 2008 to 2014 [29].

All-cause OM was based on the study from Tong et al. (2018) [7]. Rates were adapted to account for underestimation by a factor of 150%, consistent with the approach used by Stoecker et al. (2021) [7,37].

**Case fatality rates.** Age-specific case fatality rates (CFRs) for IPD were based on the weighted CFR values from ABC reports from 2017 to 2019 [23,38,39]. Hospitalized CAP CFRs were extracted from a recent cost-effectiveness study, which reported 2018 to 2019 CFR averages based on the NIS data [24].

**Vaccine effectiveness.** Estimates for vaccine effectiveness (VE) on IPD [30], CAP [31,32], and OM [33] were extrapolated from established PCV13 effectiveness and PCV7 efficacy studies and adjusted for 2019 serotype coverage of PCV15 and PCV20. Consistent with other recent cost-effectiveness studies, the direct effect was assumed to be 75.6% to account for the fact that the full VE may not be achieved until the final dose of the vaccine schedule [13,24].

**Medical costs.** Direct medical costs related to the treatment of IPD [34], CAP [34], and OM [7] were extracted from the literature and inflated to 2024 US dollars [26].

**Utilities.** Baseline utilities from the general population were sourced from the Melegaro et al. (2004) study [35].

### 2.3. Analyses

We compared PCV20 to PCV15, as both vaccines are currently recommended for US infants [10,11], and calculated their expected reduction in disease cases (IPD, inpatient CAP, outpatient CAP, and OM), as well s in disease specific costs, deaths, LYs, and QALYs. Outcomes in our analyses, including Lys and QALYs, were not discounted.

**Figure 2:**
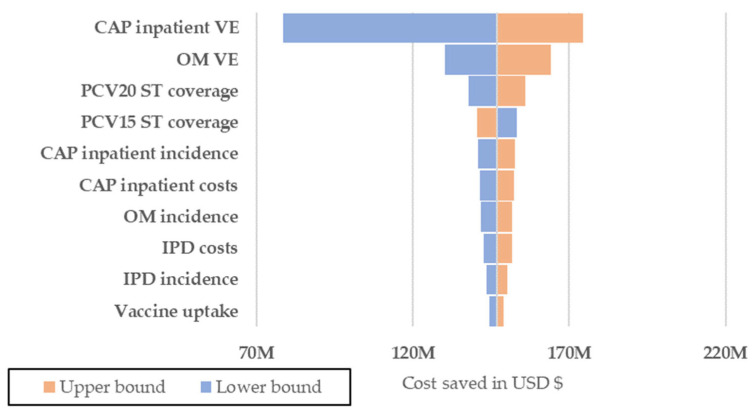
Results of the Deterministic Sensitivity Analysis (DSA)–PCV20 vs. PCV15 costs averted.

**Figure 3:**
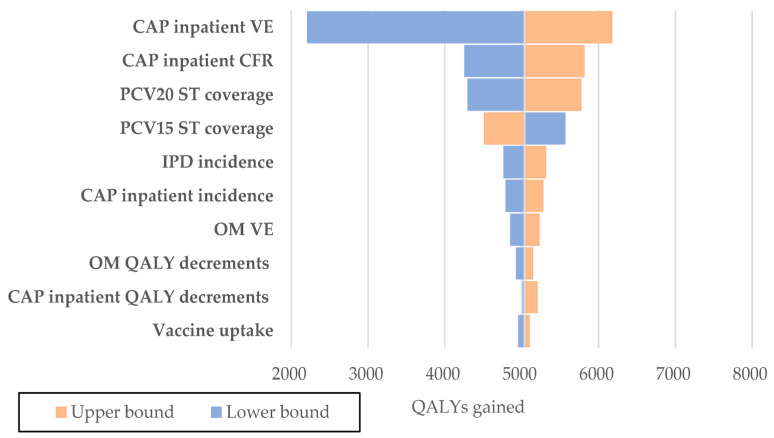
Results of the Deterministic Sensitivity Analysis (DSA)–PCV20 vs. PCV15 QALYs gained.

One-way deterministic sensitivity analyses (DSAs) were conducted to determine the impact of varying individual data inputs on the results. When available, parameters were adjusted within their reported 95% confidence intervals. If 95% were not provided, standard error (SE) was used to calculate the upper and lower bounds of the 95% confidence interval (CI). If the SE was not reported, we estimated it using the mean and sample size. For data for which no 95% CI or SE was available, we assumed the upper and lower bound to be 20% of the mean. This approach allowed us to systematically assess the robustness of our findings by quantifying the effects of parameter uncertainty. The results of these analyses are presented in tornado diagrams (Figure 2 and Figure 3), which illustrate the influence of each parameter on the overall direct costs averted and QALYs gained.

Additionally, we have included the best- and worst-case scenarios to enhance the robustness of the cost-effectiveness evaluation of PCV20 in our analysis. For the two scenarios, we utilized the DSA-low and DSA-high values.

## 3. Results

Over the course of five years, the implementation of PCV20 for newborns in the US, in comparison to PCV15, is projected to result in a significant reduction in pneumococcal disease. Specifically, an additional 220 cases of IPD will be prevented, including 47 meningitis and 172 bacteremia cases. Furthermore, the adoption of PCV20 is expected to avert 6542 cases of CAP, with 3870 requiring hospitalization and 2672 being managed on an outpatient basis. Finally, an additional decrease of 112,095 cases of OM is projected.

The implementation of PCV20 is also associated with a decrease in mortality, with 66 fewer deaths expected in comparison to the use of PCV15. The health benefits of PCV20 are considerable, yielding an additional 5058 LYs and 5037 QALYs. From an economic standpoint, PCV20 was estimated to save approximately USD 147 million more than when compared to PCV15 over 5 years for five birth cohorts. Detailed clinical and economic outcomes are provided in Table 2, including the overall results for PCV15 and PCV20, as well as the incremental results comparing PCV20 versus PCv15.

Deterministic one-way sensitivity analyses showed that the most influential model parameters impacting total incremental direct costs were CAP inpatient VE, OM VE, PCV20 and PCV15 serotype coverage and CAP inpatient incidence (Figure 2). Similarly, for QALYs gained, the main impacting drivers included CAP inpatient VE, CAP inpatient CFR, PCV20 and PCV15 serotype coverage, and IPD incidence (Figure 3).

The result of the two scenario analyses, a worst-case and best-case analysis, are shown in Table 3 and Table 4. The worst-case scenario showed that PCV20 was estimated to avert 70,325 disease cases, 15 deaths, corresponding to a gain of 1135.0 LYs and 1274.9 QALYs compared to PCV15 (Table 3). Additionally, it was estimated that PCV20 would save USD 48 million more than PCV15. The best-case scenario showed that PCV20 would avert 176,526 disease cases, 118 deaths corresponding to a gain of 9119.4 Lys, and 9320.2 QALYs compared to PCV15 (Table 4). Additionally, it was estimated that PCV20 would save approximately USD 228 million more than PCV15.

## 4. Discussion

This study estimates the health and economic effects of PCV15 and PCV20 among US children during their first year of life. The analyses showed that, compared to PCV15, PCV20 could prevent an estimated 118,856 pneumococcal cases and 66 deaths, corresponding to net gains of 5058 LYs and 5037 QALYs. Monetary savings from averted cases were estimated at USD 147 million over 5 years from five birth cohorts. Findings from deterministic sensitivity analyses and worst- and best-case scenario analyses were consistent with those from the base-case analyses. These additional health gains of PCV20 over PCV15 were driven by the broader serotype coverage of PCV20 (55.4%) versus PCV15 (39.8%).

This study represents the first assessment of the health and economic effects of a 3-dose infant series of PCV15 and PCV20 within the first year of life in the US. However, it aligns with the findings of several other studies that have consistently demonstrated the superior health benefits and cost-effectiveness of PCV20 over PCV15, including studies performed in different countries such as Argentina, Canada, Germany, Greece, Japan, South Africa, and the Republic of Korea [15,16,17,18,19,20,21,22]. Indeed, a recent systematic literature review concluded that switching from PCV13 to PCV20 would be more beneficial in terms of direct cost reduction and yield a bigger gain in QALYs compared to switching from PCV13 to PCV15; the review also concluded that the utilization of PCV20 is associated with noteworthy savings in indirect costs attributed to productivity loss in contrast to the employment of PCV15 [22].

The main difference between previously conducted cost-effectiveness studies and our current study is that the previous studies looked at population-wide effects, including indirect effects, while our study specifically focused on the direct effects on those aged less than 1 year, when children are at the highest risk of IPD, CAP, and OM. The current study isolated the direct benefits of pre-booster vaccination, providing a clearer understanding of the effects of the infant vaccination series without the confounding influence of herd effects.

Our comprehensive approach to sensitivity analysis encompasses both one-way and multivariate methods. The one-way sensitivity analysis evaluates the impact of uncertainty associated with individual parameters, while the multivariate scenarios—encompassing best-case and worst-case conditions—explore the range of potential outcomes that could result from various combinations of input variables. Notably, our conclusions remain consistent across all analyses—showing that PCV20 consistently yielded a substantially greater health and economic return compared to PCV15—demonstrating the robustness of our findings even when considering the extremes of the worst-case scenario. This consistency underlines the strength of our conclusions, which are upheld under a spectrum of different assumptions.

As PCV15 and PCV20 are newly introduced vaccines, there are no real-world data on their health impacts. Additionally, due to ethical considerations, no clinical efficacy studies were performed for these vaccines. Therefore, since there are no direct VE data for PCV15 and PCV20 available, these data were derived from studies on lower-valent pneumococcal conjugated vaccines, which is a common approach used in vaccine cost-effectiveness studies [13,15,19]. Although the data used to inform the model parameters were the most robust and up-to-date available, these assumptions introduce uncertainty into the analysis and could affect the accuracy of the health and economic projections, emphasizing the need for further clinical studies that will be necessary to validate these findings. Additionally, we made an assumption about protection during the first year of life, positing that the VE would be 75.6% of the assumed full VE. This aligns with assumptions made by others [13,24] for the VE during the first year of life and was informed by a study by Whitney et al. (2006) [40]. Another limitation is that vaccine uptake was based on estimates from the National Immunization Survey [25], which, despite potential response and recall biases, represent the best data currently available.

Finally, the cycle length of the model was one year. While this approach provided valuable insights, a more granular per-month cycle length could yield a more precise understanding of PCVs. Unfortunately, to our knowledge, data on monthly epidemiological data (such as disease incidence, serotype distribution, vaccine effectiveness, etc.) are not currently available, which restricted our ability to perform such analyses.

Given that this analysis is conducted from the healthcare perspective, which primarily addresses direct medical costs while overlooking broader societal impacts, such as productivity losses, the analysis may underestimate the overall value of PCV15 and PCV20. Consequently, while the findings indicate substantial additional health and economic outcomes of PCV20 over PCV15 from the healthcare perspective, this may represent a conservative estimate, as the true incremental benefits of PCV20 over PCV15 could be even more favorable if wider societal implications were included.

## 5. Conclusions

This study suggests that using PCV20 in the infant series would yield a significantly greater health and economic return compared to the PCV15 due to the five additional serotypes covered by PCV20.

## Figures and Tables

**Table 1:** Input parameters.

Input	Value [Upper and Lower Bound]	Reference
Population (# of births in 2023)		
Birth cohort 1 population	3,613,647	[13,27]
Birth cohort 2 population	3,552,215	[2,13]
Birth cohort 3 population	3,491,827	[13,27]
Birth cohort 4 population	3,432,466	[13,27]
Birth cohort 5 population	3,374,114	[13,27]
Life expectancy (years)	77.5	[28]
Vaccine uptake	87.5 (86.1–88.7)	[25]
Incidence (per 100 k)		
IPD	13.7 (10.1–17.4)	[13,23]
Hospitalized pneumonia	684 (635–733)	[13,24]
Non-hospitalized pneumonia	2007 (1939.5–2016.0)	[13,29]
AOM	64,770 (58.71–293)	[7,13]
Case fatality rate		
IPD	7% (6.9–7.1)	[13,23]
Inpatient CAP	1.3% (1.0–1.6)	[13,24]
PCV15 VE		
IPD	67.3% (61.5–70.5)	[13,30]
Hospitalized pneumonia	9.4% (1.6–12.6)	[13,31]
Non-hospitalized pneumonia	2.2% (−0.6–4.1)	[13,32]
AOM	2.9% (1.9–3.9)	[13,33]
PCV20 VE		
IPD	67.3% (61.5–70.5)	[13,30]
Hospitalized pneumonia	13.1% (2.3–17.5)	[13,31]
Non-hospitalized pneumonia	3.1% (−0.8–5.7)	[13,32]
AOM	4.0% (2.7–5.4)	[13,33]
Serotype coverage for <1 year		
PCV15	39.8% (31.8–47.7)	[13]
PCV20	55.4% (44.3–66.5)	[13]
Direct medical costs		
IPD	USD 57,925.74 (38,947.34–80,611.27)	[13,34]
Hospitalized pneumonia	USD 21,318.34 (19,929.57–22,753.23)	[13,34]
Non-hospitalized pneumonia	USD 717.30 (707.19–727.48)	[13,34]
AOM	USD 443.74 (439.63–447.88)	[7,13]
QALY decrements		
Meningitis	0.0232 (0.0–0.111)	[35]
Bacteremia	0.0079 (0.0–0.0291449)	[35]
Hospitalized pneumonia	0.006 (0.0–0.05)	[35]
Non-hospitalized pneumonia	0.004 (0.003–0.005)	[35]
AOM	0.005 (0.004–0.006)	[35]

**Table 2:** Overall results of five birth cohorts.

Prevented Disease Cases	PCV15	PCV20	PCV20 vs. PCV15
IPD	561	780	220
Meningitis	121	169	47
Non-meningitis	439	612	172
CAP	16,690	23,232	6542
CAP (inpatient)	9874	13,744	3870
CAP (outpatient)	6817	9489	2672
OM	285,985	398,080	112,095
**Total**	**303,236**	**422,092**	**118,856**
**Prevented deaths**			
IPD	39	55	15
Meningitis	8	12	3
Non-meningitis	31	43	12
CAP	128	179	50
**Total**	**168**	**233**	**66**
**Direct costs saved**			
IPD	USD 32,470,382	USD 45,197,466	USD 12,727,084
CAP (inpatient)	USD 210,486,814	USD 292,989,183	USD 82,502,369
CAP (outpatient)	USD 4,889,648	USD 6,806,193	USD 1,916,545
OM	USD 126,904,740	USD 176,646,296	USD 49,741,556
**Total**	**USD 374,751,584**	**USD 521,639,139**	**USD 146,887,555**
**LYG**			
IPD deaths	3021.4	4205.6	1184.3
Meningitis	652.6	908.4	255.8
Non-meningitis	2368.8	3297.2	928.5
CAP deaths	9883.4	13,757.3	3873.9
**Total**	**12,904.8**	**17,962.9**	**5058.1**
**QALYs gained**			
IPD cases	6.3	8.7	2.5
Meningitis	2.8	3.9	1.1
Non-meningitis	3.5	4.8	1.4
OM cases	1429.9	1990.4	560.5
CAP cases (inpatient)	59.2	82.5	23.2
CAP cases (outpatient)	27.3	38.0	10.7
IPD deaths	2651.9	3691.4	1039.5
CAP deaths	8674.9	12,075.1	3400.2
**Total**	**12,849.6**	**17,886.1**	**5036.5**

**Table 3:** Worst-case scenario results of five birth cohorts.

Prevented Disease Cases	PCV15	PCV20	PCV20 vs. PCV15
IPD	297	414	117
Meningitis	64	89	25
Non-meningitis	233	324	92
CAP	−222	−137	85
CAP (inpatient)	1528	2196	668
CAP (outpatient)	−1750	−2333	−583
OM	166,542	236,665	70,123
**Total**	**166,617**	**236,942**	**70,325**
**Prevented deaths**			
IPD	20	29	8
Meningitis	4	6	2
Non-meningitis	16	22	6
CAP	15	22	7
**Total**	**36**	**51**	**15**
**Direct costs saved**			
IPD	USD 11,567,811	USD 16,114,906	USD 4,547,096
CAP (inpatient)	USD 30,447,049	USD 43,767,632	USD 13,828,137
CAP (outpatient)	USD −1,237,459	USD −1,649,945	USD −412,486
OM	USD 73,216,825	USD 104,044,962	USD 30,828,137
**Total**	**USD 113,994,226**	**USD 162,277,556**	**USD 48,283,330**
**LYG**			
IPD deaths	1578.0	2198.3	620.3
Meningitis	340.9	474.8	134.0
Non-meningitis	1237.2	1723.5	486.3
CAP deaths	1176.4	1691.0	514.7
**Total**	**2754.4**	**3889.3**	**1135.0**
**QALYs gained**			
IPD cases	0.0	0.0	0.0
Meningitis	0.0	0.0	0.0
Non-meningitis	0.0	0.0	0.0
CAP cases (inpatient)	0.0	0.0	0.0
CAP cases (outpatient)	−5.2	−7.0	−1.7
OM cases	666.2	946.7	280.5
IPD deaths	1385.1	1929.5	544.5
CAP deaths	1032.5	1484.2	451.7
**Total**	**3078.5**	**4353.4**	**1274.9**

**Table 4:** Best-case scenario results of 5 birth cohorts.

Prevented Disease Cases	PCV15	PCV20	PCV20 vs. PCV15
IPD	849	1264	414
Meningitis	183	273	89
Non-meningitis	666	991	325
CAP	27,111	37,672	10,561
CAP (inpatient)	14,307	19,871	5564
CAP (outpatient)	12,804	17,801	4997
OM	430,433	595,984	165,551
**Total**	**458,393**	**634,919**	**176,526**
**Prevented deaths**			
IPD	60	90	29
Meningitis	13	19	6
Non-meningitis	47	70	23
CAP	229	318	89
**Total**	**289**	**408**	**118**
**Direct costs saved**			
IPD	USD 68,472,421	USD 101,866,130	USD 33,393,709
CAP (inpatient)	USD 325,530,443	USD 452,125,615	USD 126,595,172
CAP (outpatient)	USD 9,314,713	USD 12,949,723	USD 3,635,010
OM	USD 192,782,182	USD 266,929,175	USD 74,146,993
**Total**	**USD 596,099,758**	**USD 833,870,642**	**USD 237,770,884**
**LYG**			
IPD deaths	4643.8	6908.5	2264.7
Meningitis	1003.1	1492.2	489.2
Non-meningitis	3640.7	5416.3	1775.6
CAP deaths	17,626.2	24,480.9	6854.6
**Total**	**22,270.0**	**31,389.4**	**9119.4**
**QALYs gained**			
IPD cases	39.8	59.2	19.4
Meningitis	20.4	30.3	9.9
Non-meningitis	19.4	28.9	9.5
CAP cases (inpatient)	715.3	993.5	278.2
CAP cases (outpatient)	64.0	89.0	25.0
OM cases	2582.6	3575.9	993.3
IPD deaths	4075.9	6063.8	1987.8
CAP deaths	15,471.0	21,487.5	6016.5
**Total**	**22,948.7**	**32,268.9**	**9320.2**

## Data Availability

All data used for estimation of model parameter values may be found in the manuscript.
